# Estimation of rice seedling growth traits with an end-to-end multi-objective deep learning framework

**DOI:** 10.3389/fpls.2023.1165552

**Published:** 2023-06-02

**Authors:** Ziran Ye, Xiangfeng Tan, Mengdi Dai, Yue Lin, Xuting Chen, Pengcheng Nie, Yunjie Ruan, Dedong Kong

**Affiliations:** ^1^ Institute of Digital Agriculture, Zhejiang Academy of Agricultural Sciences, Hangzhou, China; ^2^ Institute of Spatial Information for City Brain (ISICA), Hangzhou City University, Hangzhou, China; ^3^ Institute of Agricultural Bio-Environmental Engineering, College of Bio-systems Engineering and Food Science, Zhejiang University, Hangzhou, China; ^4^ Academy of Rural Development, Zhejiang University, Hangzhou, China

**Keywords:** growth traits, fresh weight, rice seedling, deep learning, convolution neural network

## Abstract

In recent years, rice seedling raising factories have gradually been promoted in China. The seedlings bred in the factory need to be selected manually and then transplanted to the field. Growth-related traits such as height and biomass are important indicators for quantifying the growth of rice seedlings. Nowadays, the development of image-based plant phenotyping has received increasing attention, however, there is still room for improvement in plant phenotyping methods to meet the demand for rapid, robust and low-cost extraction of phenotypic measurements from images in environmentally-controlled plant factories. In this study, a method based on convolutional neural networks (CNNs) and digital images was applied to estimate the growth of rice seedlings in a controlled environment. Specifically, an end-to-end framework consisting of hybrid CNNs took color images, scaling factor and image acquisition distance as input and directly predicted the shoot height (SH) and shoot fresh weight (SFW) after image segmentation. The results on the rice seedlings dataset collected by different optical sensors demonstrated that the proposed model outperformed compared random forest (RF) and regression CNN models (RCNN). The model achieved R^2^ values of 0.980 and 0.717, and normalized root mean square error (NRMSE) values of 2.64% and 17.23%, respectively. The hybrid CNNs method can learn the relationship between digital images and seedling growth traits, promising to provide a convenient and flexible estimation tool for the non-destructive monitoring of seedling growth in controlled environments.

## 1 Introduction

Plant factories achieve stable and efficient growing of plants by controlling the growing environment (Ares et al., 2021; McClements et al., 2021). Various plant factories have been promoted in China to cope with the shortage of cultivated lands for vegetable production. In recent years, industrial rice seedlings have attracted attention because of good economic benefits (Ma et al., 2014). At present, the rice seedlings bred in the factory need to be selected manually and then transplanted into the field. Plant growth is a response to environmental parameters (Chen et al., 2016). Plant phenotype is the character of plants under the interaction between intrinsic genotype and external environmental conditions (Furbank and Tester, 2011). Phenotypic morphological traits such as height, leaf area and biomass, can be obtained by measurement and weighing, which is helpful for quantifying plant growth (Watt et al., 2020). The traditional methods of manual trait measurement are simple and accurate, but they are difficult to meet the demand of high-throughput trait acquisition in large quantities, and usually require destructive sampling, which is time-consuming and laborious (Hüther et al., 2020). The estimation of plant growth is a non-negligible element in the intelligent development of plant factories; thus it is of great practical significance to develop rapid, accurate and automatic methods for obtaining plant growth-related traits to replace some tedious manual measurements.

The development of computer vision provides a good opportunity for image-based automatic measurement and acquisition of plant phenotype data. Mortensen et al. (2018) proposed a method for segmenting lettuce in 3D point clouds and estimating their yield. Reyes-Yanes et al. (2020) used MASK-RCNN to segment the lettuce from the background and used the geometric features extracted from the segmented data to build a fresh weight regression model. Nowadays, RGB images can be obtained at a low cost by using sensors such as digital cameras and smartphones, which are affordable and easy to operate. Computer vision algorithms are then used to extract image-based phenotypic data and apply them to downstream tasks. For example, Yu et al. (2013) proposed a crop segmentation method and used the skeleton endpoint to characterize the leaf of the seedling to recognize the growth stage of the seedling. Borianne et al. (2018) developed software for automatic cereal root system measurements from digital images. These works show that images are promising to provide a non-destructive and convenient access to obtain plant growth information, and the key is to construct appropriate feature extraction methods.

In recent years, convolutional neural networks (CNNs), an advanced deep learning method, have been widely applied to visual tasks in the field of agriculture, such as plant detection (Quan et al., 2019), classification (Perugachi-Diaz et al., 2021), segmentation (Gong et al., 2021) and counting (Osco et al., 2020; He et al., 2022). Benefiting from the ability of automatic feature learning and hierarchical feature extraction, CNNs originally designed for classification tasks can also perform well for regression tasks. Some studies have used regression CNNs for plant growth trait estimation in controlled environments, and most of them target lettuce (Chen et al., 2016; Zhang et al., 2020; Buxbaum et al., 2022; Lin et al., 2022) and Arabidopsis thaliana (Ubbens and Stavness, 2017). Existing studies illustrate that regression CNNs can model the relationship between images and growth traits of leafy vegetables well. Meanwhile, there is a great demand for automatic measurement of growth-related traits in rice seedlings (Lu et al., 2017; Mekawy et al., 2018; Zhang et al., 2019). However, applying existing regression CNNs for the growth monitoring of grain crops such as rice still needs to be further validation, as morphological differences between monocot and dicot plants exist from the seedling stage, which challenges the estimation of rice seedling growth traits directly from digital images.

The objective of this study was to accurately estimate growth-related traits of rice seedlings in controlled environment agriculture. A CNN-based framework including image preprocessing, image augmentation, semantic segmentation network and regression network, was used to segment RGB images of rice seedlings and model the relationship between the images and the corresponding growth-related traits (height and fresh weight). This study explored the potential of using CNNs with digital images to estimate growth-related traits of rice seedlings in vertical planting modules to establish a feasible and robust seedling growth monitoring method.

## 2 Material and methods

### 2.1 Image collection and preprocessing

The rice cultivar ZY-18 (Zheyou 18, hybrid indica) was selected for experiments. ZY-18 was bred by the Zhejiang Academy of Agricultural Sciences (ZAAS) and has been widely planted in Zhejiang and surrounding provinces in China. The seeds used in this study were obtained from the market. The rice seedlings used in this work were grown in a vertical growth unit in a laboratory in Binjiang, Hangzhou (N30°11′, E120°12′). After surface disinfection, rice seeds were germinated in dark for two days and then sowed into substrate trays. Rice seedlings were grown under controlled climate conditions, with day/night temperatures of 26-28°C/18-20°C and average relative humidity of 75%. During the seedling growth period, full spectrum led grow lights were used for illumination at a light intensity of 400 µmol·m-2·s-1 and 14 to 16 hours during the day. The experiment was performed from November 20, 2021, to December 16, 2021.

A Nikon Z5 camera and a smartphone (iPhone 12) were used for image acquisition, in which the Nikon camera shot 10 seedlings at a time and the iPhone shot one seedling at a time. During the image collection, the sensors were placed on the top of a photography light box (60×60×60 cm) to capture digital images. According to the difference in the sensor size, the resolution of the original digital image is 4016×6016 (Nikon Z5) and 3024×4032 (iPhone), respectively. All digital images are stored in PNG format. Finally, two datasets were constructed, a digital image dataset containing 92 images captured by a digital camera, and a digital image dataset containing 76 images captured by a smartphone.

For the digital images of seedlings in both datasets, binarized labels consistent with the original image size were generated by manual annotation. The rectangle enclosing a single seedling was generated using binarized labels, and the original images were cropped using these rectangles. A total of 984 rice seedling images were obtained as the new dataset.

Referring to the previous study, the image dataset was randomly divided into a training set and a test set in a ratio of 8:2, which were used to construct and evaluate the model, respectively. Further, 20% of the training dataset was randomly selected as the validation set during training. To prevent overfitting, data augmentation was performed on the fly when training all models, which consisted of horizontal flipping, horizontal shift and random rotation.

### 2.2 Measurement of traits

Manual measurements were taken at the same time as image collection. Ten seedlings were randomly sampled from each planting tray. After washing and drying the seedlings, the plant height and fresh weight were measured using a ruler and a digital balance with a resolution of 0.1 cm and 0.0001 g, respectively. These measurements were conducted on days 10 (December 3), 17(December 10) and 22(December 15) after seeding. Finally, a regression dataset with 504 samples was obtained, in which each sample had measurements of seedling height and shoot fresh weight after root removal. Meanwhile, each seedling sample had its corresponding binarized label in the segmentation dataset above.

### 2.3 Construction of the network architecture

**Figure 1:**
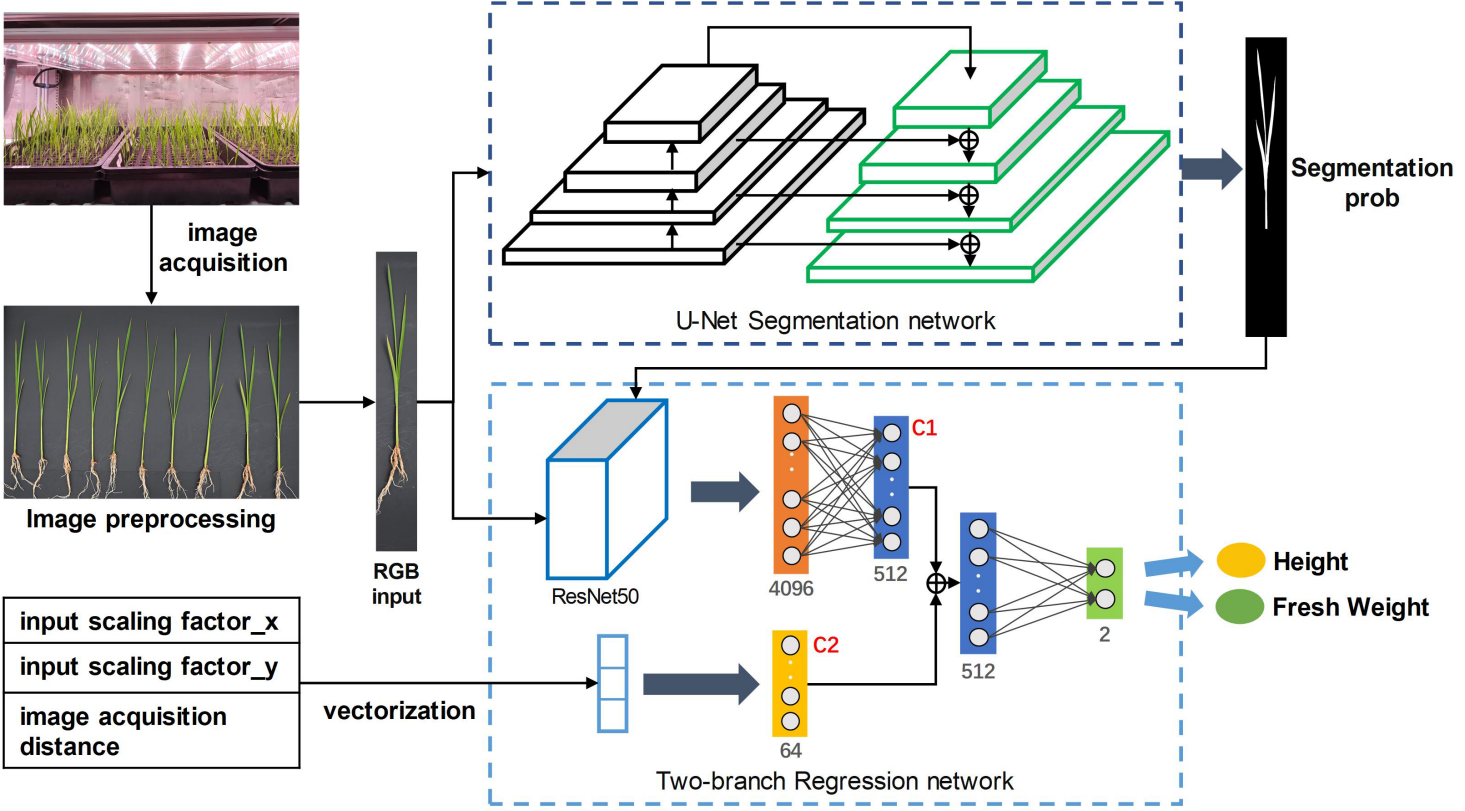
Overall structure of the hybrid CNN framework.

The whole process is shown in **Figure 1**. In the first stage, the RGB image of rice seedlings with the size of 512×512×3 was input into the U-Net (Ronneberger et al., 2015) segmentation model to output the probability map of rice seedlings. In the second stage, based on the pre-experiment, a modified ResNet50 (He et al., 2016) was used as the backbone of the regression network to predict growth-related traits. Specifically, the RGB image of rice seedlings and segmentation predictions above were concatenated as the input of the feature extraction network. Besides, a branch fully connected layer was introduced to receive a geometric vector including the input scaling factor and image acquisition distance as the input. The fully connected layers of the two paths were fused into a feature vector of 576×1 at the depths of the network. And this feature vector was passed to the regression head, which consisted of two fully connected layers. Finally, the regression network output two values, which represented the prediction results of seedling height and fresh weight.

In the training phase, the Adam optimizer was used to optimize the parameters of the two networks in stages. In the beginning, only the parameters of the segmentation network were updated. After 30 epochs, the segmentation model reached convergence and the parameters were frozen. Subsequently, the parameters of the regression network were updated until the model converged. The initial learning rate was set to 0.001, batch size was set to 4, and the training period was set to 300 epochs. The loss function of segmentation and regression were cross entropy loss and smooth L1 loss, respectively. The “ReduceLROnPlateau” scheduler and “Early Stopping” strategy was adopted to adjust the learning rate and control the training process: If the validation loss did not improve within 50 epochs, the learning rate will decrease by 0.1 times. If the validation loss still did not improve within 100 epochs, the training will be terminated.

### 2.4 Performance evaluation

To evaluate the proposed model, overall accuracy (OA), F1-score and Intersection-over-Union (IoU) metrics were used as the criteria for segmentation. Mean absolute error (MAE), normalized root mean square error (NRMSE) and coefficient of determination (R^2^) were calculated to evaluate the estimation performance. These metrics are defined as follows:


(1) $$\text{OA}=\frac{TP+TN}{TP+TN+FP+FN}$$

(2) $$\text{Precision}=\frac{TP}{TP+FP},\text{ Recall}=\frac{TP}{TP+FN}$$

(3) $$\text{F}1=2\times \frac{Precision\times Recall}{Precision+Recall}$$

(4) $$\text{IoU}=\frac{TP}{FP+TP+FN}$$

where TP is the number of correctly classified seedling pixels, FP is the number of pixels misclassified as seedlings, FN is the number of pixels misclassified as background, TN is the number of correctly classified background pixels.


(5) $$\text{MAE}=\frac{1}{n}\sum_{i=1}^{n}\left|y_{i}-f_{i}\right|$$

(6) $$\text{NRMSE}=\frac{\sqrt{\frac{1}{n}\sum_{i=1}^{n}{\left(y_{i}-f_{i}\right)}^{2}}}{\bar{y}}$$

(7) $$R^{2}=1-\frac{\sum_{i=1}^{n}{\left(y_{i}-f_{i}\right)}^{2}}{\sum_{i=1}^{n}{\left(y_{i}-\bar{y}\right)}^{2}}$$

where n is the number of samples, $f_{i}$ is the *i-th* predicted trait, $y_{i}$ is the *i-th* ground truth trait, $\bar{y}$ is the average of ground truth.

To further evaluate the estimation performance of the proposed model, the classical machine learning classifier RF (Breiman, 2001) and a regression CNN model (RCNN) were adopted to estimate the growth traits of rice seedlings. RF has shown good performance in the estimation of growth traits of crops and fishes (Saberioon and Císař, 2018; Zhang et al., 2020), while RCNN has been reported in estimating the fresh weight of lettuce directly from the input images (Zhang et al., 2020). To build RF classifier, feature extraction was conducted from digital images of rice seedlings. According to the characteristics of seedlings, low-level image features including color features, morphological features and texture features were extracted. **Table 1** lists all the features used to build the RF model. Because the RF model itself can evaluate the importance of features, all low-level features were used to fit the RF model in the experiment.

**Table 1:** List of image features of rice seedlings.

No.	Feature	Description	Symbols and formulas
1	Average	The average of each color component in five color spaces (RGB, HLS, HSV, CIELab, YCbCr)	*Ave*
2	Standard deviation	The standard deviation of each color component in 5 color spaces (RGB, HLS, HSV, CIELab, YCbCr)	σ
3	Area	The number of pixels in the seedling area	*A*
4	Perimeter	The number of pixels of the seedling boundary	*P*
5	Aspect ratio	Ratio of length to width of minimum rectangle of seedling	Ar=LW
6	Ellipse ratio	Ratio of the major axis to the minor axis of the seedling equivalent ellipse	Er=aellipsebellipse
7	Compactness	Ratio of the diameter of the minimum enclosing circle to the length of the minimum rectangle	Cpa=2rL
8	Arc	Ratio of the seedling area to the area of the minimum enclosing circle	Arc=Aπr2
9	Extent	Ratio of the seedling area to the area of the minimum rectangle	Ex=AL×W
10	Complexity	Ratio of the square of the seeding perimeter to the seedling area	Cpl=P2A
11	Contrast	Contrast of the gray level co-occurrence matrix in seedling region	
12	Correlation	Correlation of the gray level co-occurrence matrix in seedling region	
13	Energy	Energy of the gray level co-occurrence matrix in seedling region	
14	Homogeneity	Homogeneity of the gray level co-occurrence matrix in seedling region	

### 2.5 K-fold cross validation for regression

The regression dataset comprises 504 images, which is a relatively small dataset in the deep learning community. As mentioned in the part of data preprocessing above, 80% of the samples were randomly selected for modeling, and the remaining 20% were used for evaluation. To prevent overfitting, K-fold cross-validation (K=5) was used to build the model on the training set (Stone, 1974). The average of metrics on the test set was taken as the evaluation standard.

## 3 Results

### 3.1 Segmentation results of the model

**Figure 2:**
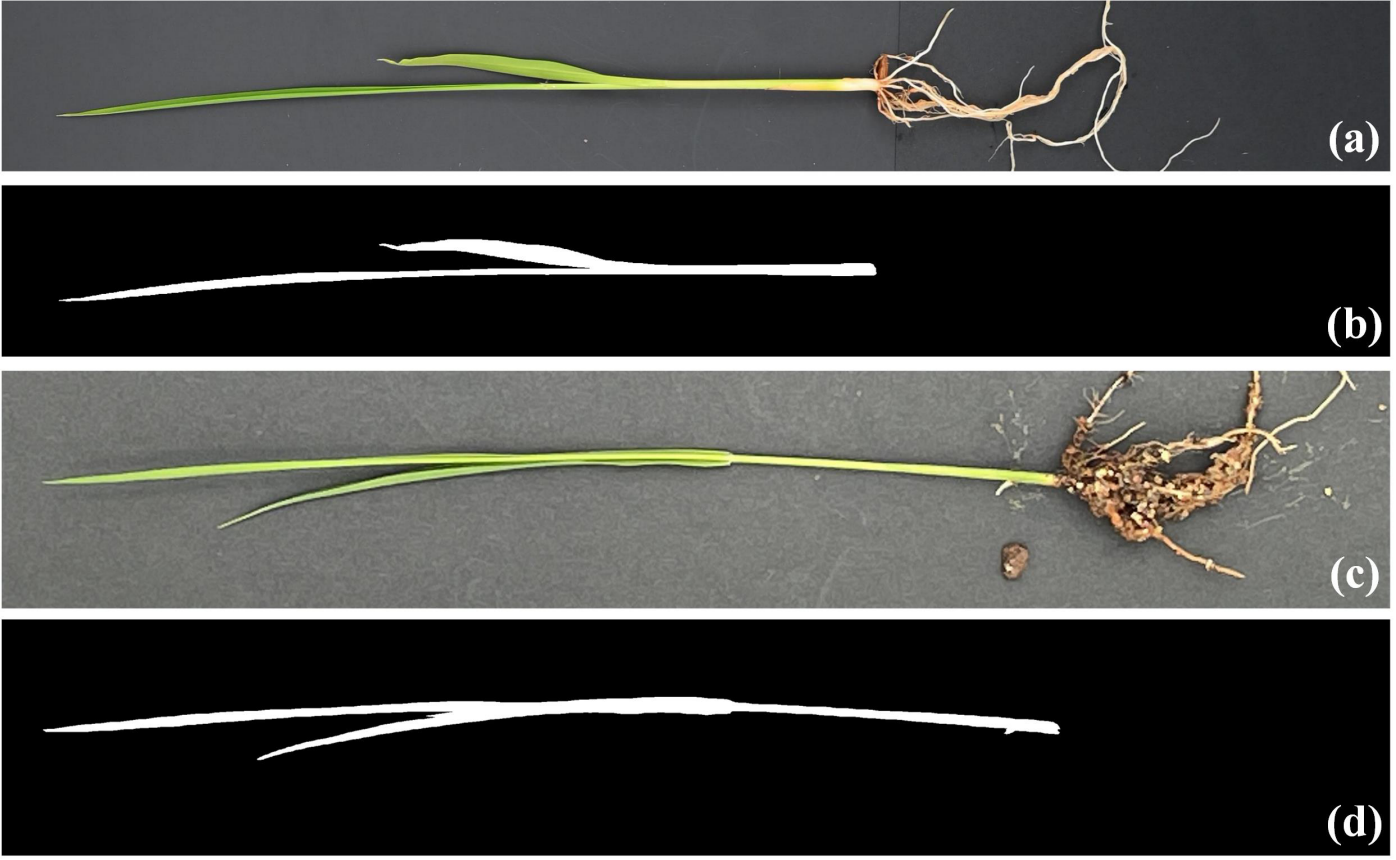
Example of results on the segmentation test set **(A, C)** shows the original image taken with a digital camera and a smartphone, respectively, and **(B, D)** shows the corresponding segmentation results.

As demonstrated by the accuracy evaluation of the proposed method on the rice seedling segmentation task, the segmentation submodel achieved an OA of 0.997, an F1 accuracy of 0.956 and the IoU accuracy of 0.916 (**Table 2**). Visual interpretation on the test set indicated that the proposed method can distinguish seedling pixels from background pixels well (**Figure 2**). In conclusion, the model can recognize complete rice seedlings with high accuracy.

**Table 2:** Confusion matrix of segmentation test set.

Prediction		
Ground truth	Rice	Background
Rice	3,428,953	165,599
Background	146,830	98,088,319
OA	0.997	
F1	0.956	
IoU	0.916	

### 3.2 Estimation results of the model

**Figure 3:**
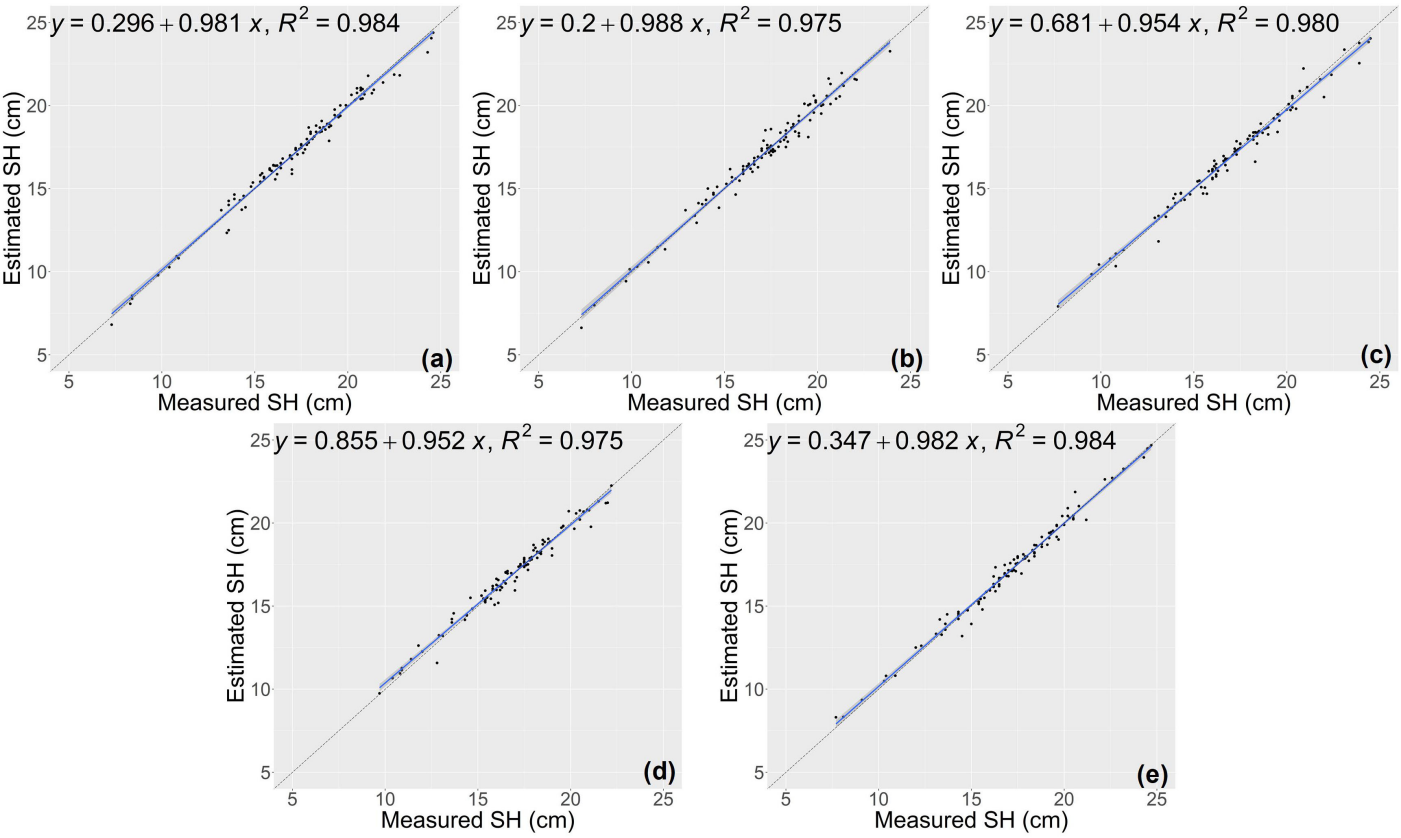
Estimation results of shoot height (SH) based on the proposed model **(A–E)** shows the results in the five-fold cross-validation.

**Figure 4:**
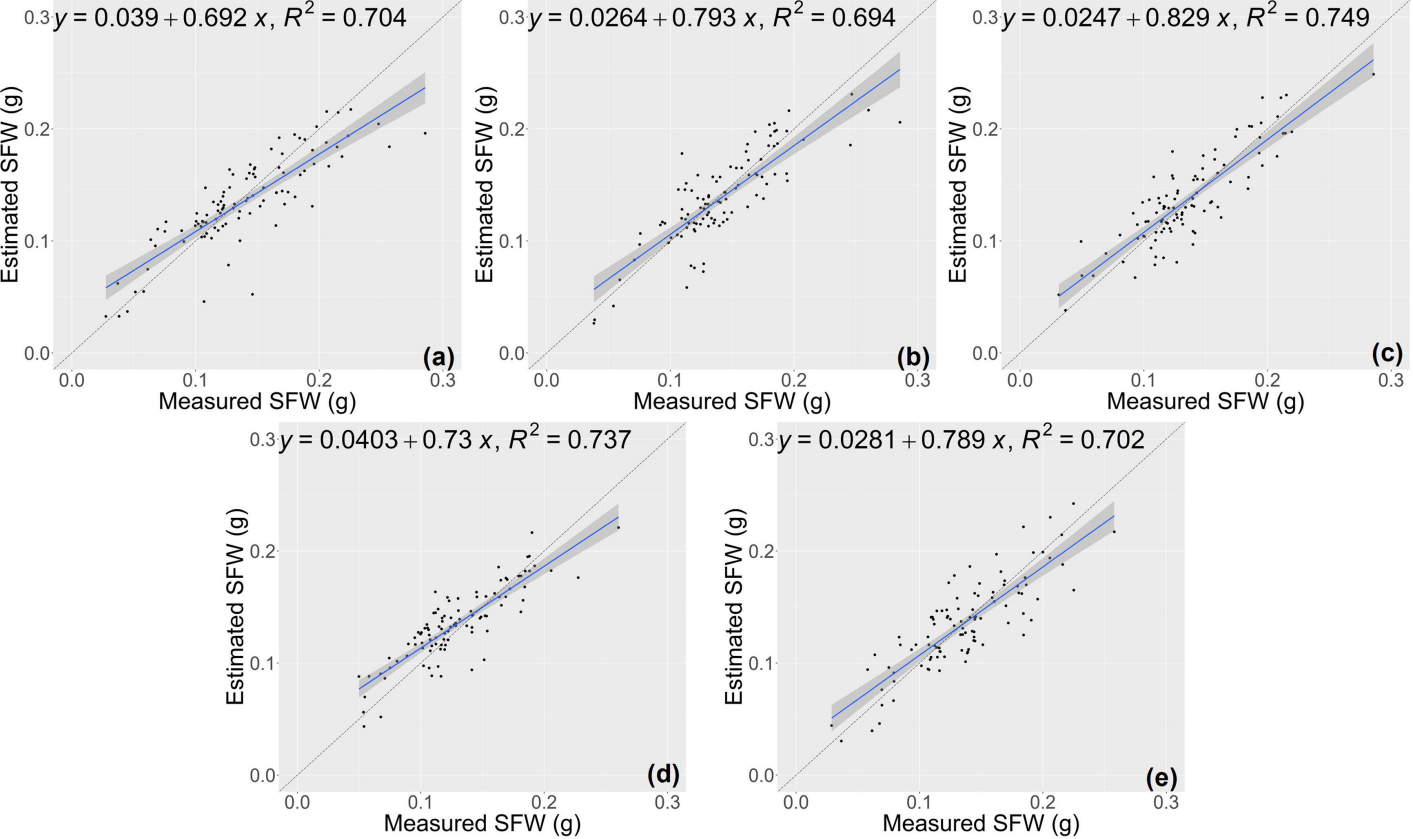
Estimation results of shoot fresh weight (SFW) based on the proposed model **(A–E)** shows the results in the five-fold cross-validation.

The results of growth-related traits estimation based on the proposed method are shown in **Figures 3** and **4**, where the figures depict the prediction results in the five-fold cross-validation. **Table 3** showed the performance of the proposed model on the test sets for estimating growth-related traits of rice seedlings. The results indicate a strong correlation between the actual measured values of rice seedling growth-related traits and the CNN-based model estimates. In terms of height traits, the regression submodel had a good estimation performance with an average R^2^ of 0.980 and an average NRMSE of 2.64%. The results of seedling shoot biomass estimation were slightly worse, with an average R^2^ of 0.717 and an average NRMSE of 17.23%.

**Table 3:** Regression error statistics of the proposed method for growth-related traits.

Number of experiments	SH			SFW		
	MAE(g)↓	NRMSE(%)↓	R^2^ ↑	MAE(g)↓	NRMSE(%)↓	R^2^ ↑
1	0.338	2.52	0.984	0.020	19.51	0.704
2	0.394	2.92	0.975	0.018	17.05	0.694
3	0.346	2.83	0.980	0.018	16.06	0.749
4	0.323	2.55	0.975	0.017	16.08	0.737
5	0.290	2.35	0.984	0.019	17.44	0.702
Average	0.338	2.64	0.980	0.018	17.23	0.717

### 3.3 Comparison of the results with the conventional estimation methods

**Figure 5:**
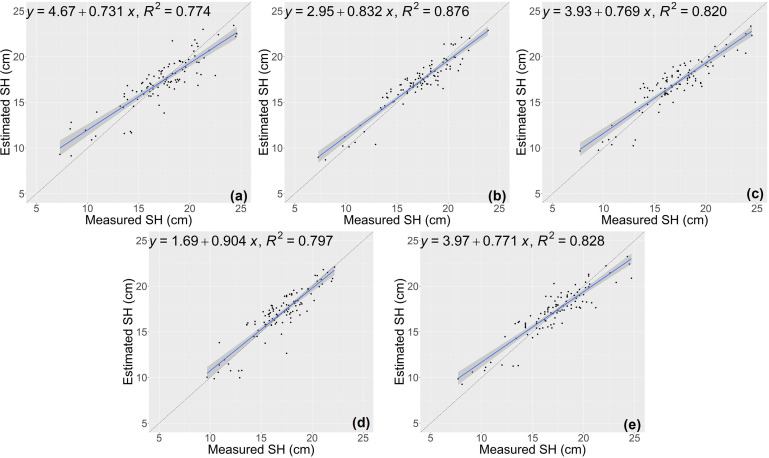
Estimation results of shoot height (SH) based on RF model **(A–E)** shows the results in the five-fold cross-validation.

**Figure 6:**
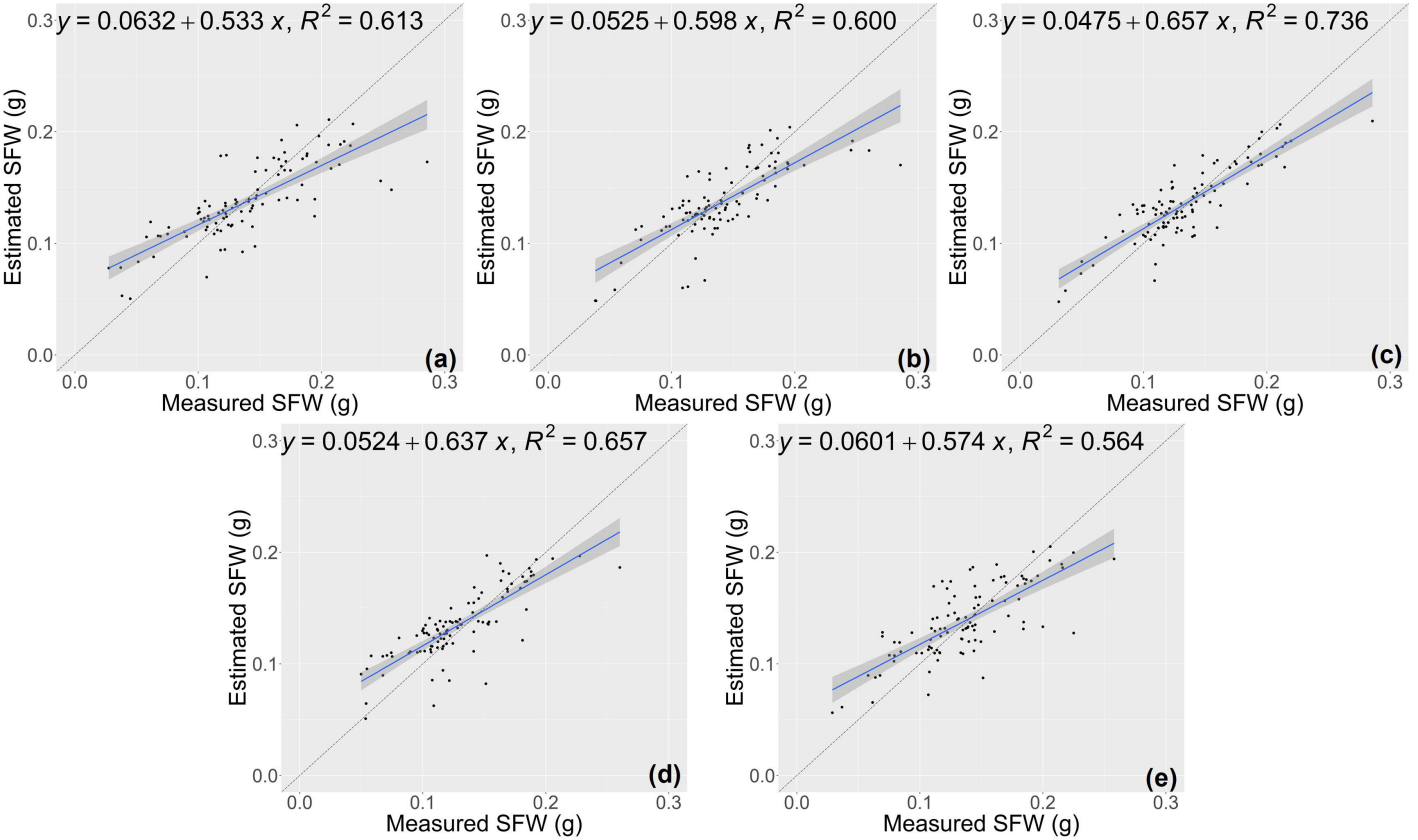
Estimation results of shoot fresh weight (SFW) based on RF model **(A–E)** shows the results in the five-fold cross-validation.

The random forest (RF) model was constructed based on the features selected above (**Table 1**), and the number of trees in the RF model was set as 1000 by grid search. The estimation results of RF classifier were shown in **Table 4**, **Figures 5** and **6**. The average R^2^ of RF for height estimation results was 0.819, with an average NRMSE of 7.93%. Similar to the results of the CNN model, the estimation performance of biomass traits was lower than that of height traits, with an average R^2^ of 0.634 and an average NRMSE of 19.41%.

**Table 4:** Regression error statistics of the RF method for growth-related traits (n=1000).

Number of experiments	SH			SFW		
	MAE(g)↓	NRMSE(%)↓	R^2^ ↑	MAE(g)↓	NRMSE(%)↓	R^2^ ↑
1	1.289	9.48	0.774	0.023	22.42	0.613
2	0.913	6.81	0.876	0.020	19.15	0.600
3	1.116	8.09	0.820	0.017	16.43	0.736
4	0.963	7.45	0.797	0.018	18.26	0.657
5	1.063	7.83	0.828	0.022	20.79	0.564
Average	1.069	7.93	0.819	0.020	19.41	0.634

**Figure 7:**
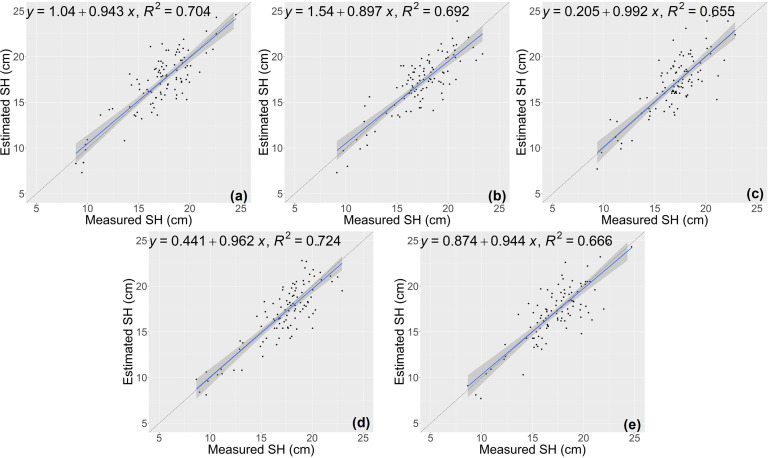
Estimation results of shoot height (SH) based on the RCNN model **(A–E)** shows the results in the five-fold cross-validation.

**Figure 8:**
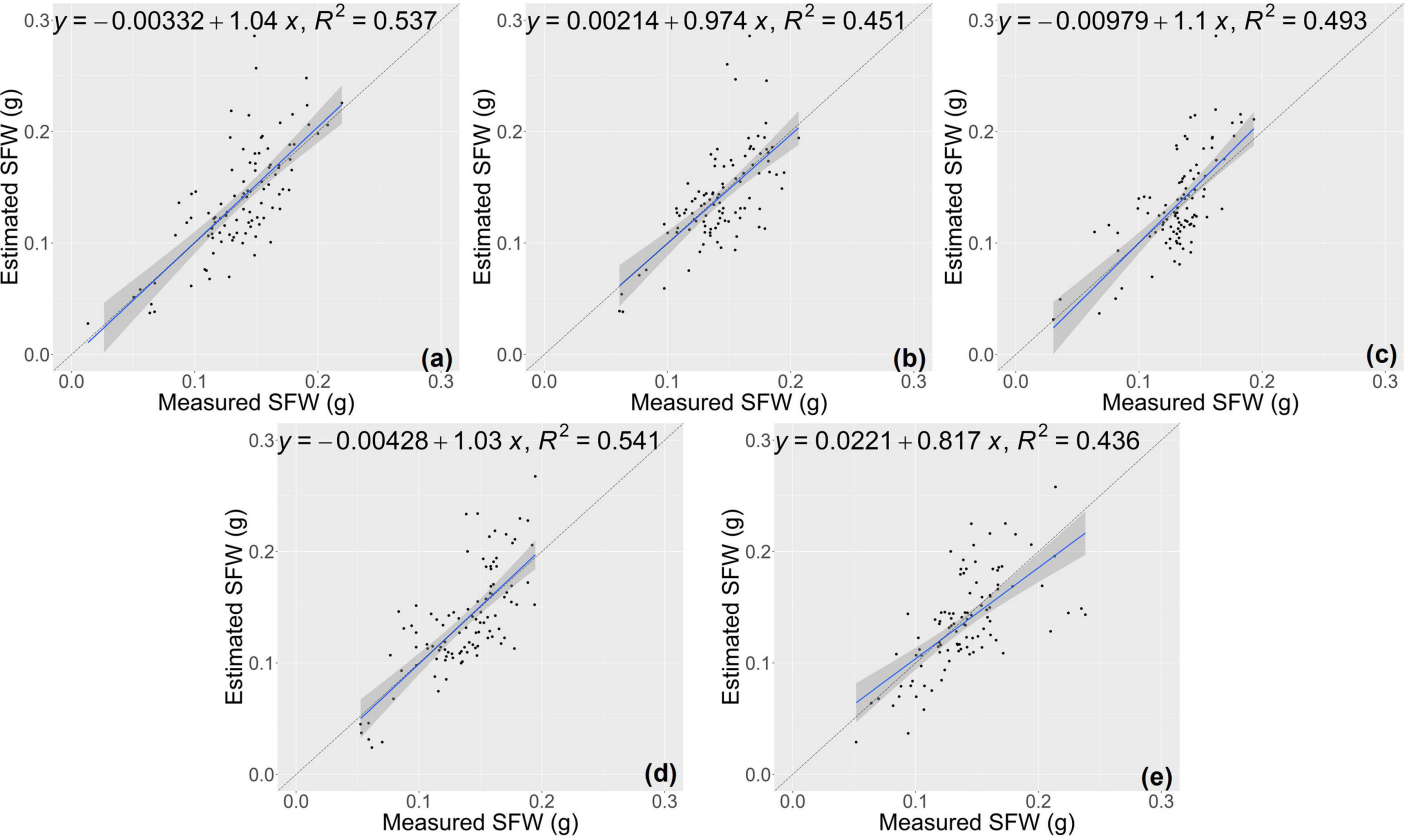
Estimation results of shoot fresh weight (SFW) based on the RCNN model **(A–E)** shows the results in the five-fold cross-validation.

The estimation results of the RCNN model were shown in **Figures 7** and **8**. For the two traits of SH and SFW (**Table 5**), the RCNN model had average R^2^ values of 0.688 and 0.492, respectively, and the average NRMSE values were 10.81%, and 23.27%, respectively. The results showed that this regression CNN model struggled in estimating seedling growth-related traits.

**Table 5:** Regression error statistics of the RCNN method for growth-related traits.

Number of experiments	SH			SFW		
	MAE(g)↓	NRMSE(%)↓	R^2^ ↑	MAE(g)↓	NRMSE(%)↓	R^2^ ↑
1	1.559	10.83	0.704	0.025	24.22	0.537
2	1.442	10.93	0.692	0.023	22.22	0.451
3	1.508	11.01	0.655	0.024	22.60	0.493
4	1.455	10.45	0.724	0.026	23.17	0.541
5	1.419	10.84	0.666	0.025	24.16	0.436
Average	1.477	10.81	0.688	0.025	23.27	0.492

## 4 Discussion

### 4.1 Comparative analysis of different models

A hybrid segmentation and regression network was built in this study. Compared with existing methods that combine threshold segmentation with deep regression networks, this work adopts a two-stage model to solve segmentation and estimation tasks in an end-to-end manner. In the segmentation stage, a deep segmentation network was used instead of the classic image segmentation algorithms (such as threshold segmentation) mainly for the consideration of computational efficiency. Since the seedling images used in this research were acquired by different sensors, using a data-driven deep segmentation network can automatically learn effective features from images, reducing the difficulty of handcrafted feature design and expert knowledge. In the regression stage, image segmentation prediction is taken as explicit input to help the regression network focus on the seedling pixel region rather than the redundant background pixels. This makes the whole model easier to be optimized on a small dataset. Furthermore, the proposed method is flexible for varying image acquisition distance since the acquisition distance and image scaling factor are taken as inputs to the regression network branches when constructing the model. Meanwhile, the model has good scalability and it can be adapted from the dual task of image segmentation and regression to only perform the segmentation or image-based growth character estimation.

In comparison with the classical machine learning methods, the estimation accuracy of the proposed CNN model for growth-related traits was higher than that of the RF estimator, as shown in **Tables 3** and **4**. The estimation accuracy of the former was 0.083 higher in R^2^ for biomass traits and 0.161 higher in R^2^ for height traits, and the estimation results of the CNN model also had lower NRMSE. The results demonstrate the advantages of the CNN model in automatically learning complex features from image data. However, the results of RCNN were somewhat counterintuitive. As can be seen from the tables above, the performance of RCNN for seedling traits estimation was lower than that of the proposed model and RF model. This finding indicates the features extracted by the RCNN are not robust enough to estimate rice seedling traits, which may be due to the capacity limitation of the five-layer network model. Another possible reason is that the dataset in this paper contains digital images collected by two sensors at different distances, which increases the difficulty of directly estimating growth traits from images.

According to the experimental results, including the proposed method and the other two comparison methods, the performance of seedling height estimation is better than that of biomass estimation. This may be because the information extracted from the plane digital images of a single perspective of rice seedlings can better reflect the characteristics of height traits. However, seedling images from a single perspective still had partial occlusion or hiding, which made the features learned by the model not comprehensive enough for estimating shoot biomass, affecting the estimation accuracy. Therefore, the estimation of height trait by the proposed model is better than that of biomass trait. In our opinion, more training samples may help to improve the performance of biomass estimation.

Nowadays, many reports have introduced image-based approaches to extract phenotypic traits from crop images, with results listed in **Table 6**. Different from previous works, this study focuses on the growth of rice seedlings in a controlled environment. Specifically, this study explores how CNN-based deep learning techniques can better cope with RGB images acquired under varying acquisition conditions (sensor and acquisition distance). Therefore, acquisition condition-related geometric vectors are considered as branch inputs of the regression network. In addition, unlike some methods that segment images and then extract geometric features, this study integrates image segmentation and image regression into a unified end-to-end framework, prompting the network to automatically learn the implicit representations in segmented images. The experimental results show that the presented approach in this paper is competent for the estimation of growth traits of rice seedlings.

**Table 6:** An overview of existing image-based methods for plant growth traits estimation.

Ref.	Plants	Input data types	Methods	Descriptions
(Chen et al., 2016)	Lettuce	RGB	Image segmentation + Linear regression	Fresh weight regression by geometric features extracted from a stereo-vision system.
(Zhang et al., 2020)	Lettuce	RGB	CNN regression	Multi-objective regression (Fresh weight, dry weight and area) by a five-layer CNN model.
(Lin et al., 2022)	Lettuce	RGB-D	CNN segmentation + CNN regression	Fresh weight regression by a ResNet34 model, fusing RGB-D features and geometric features extracted from segmented images.
(Buxbaum et al., 2022)	Lettuce	RGB-D	CNN regression	Fresh weight regression by a ResNet50 model, fusing RGB-D image features
(Ubbens and Stavness, 2017)	Arabidopsis thaliana	RGB	CNN regression	Age regression by a five-layer CNN model
(Quan et al., 2021)	Weed	RGB-D	CNN regression	Fresh weight regression by a DenseNet201 model, fusing RGB-D image features
This work	Rice seedling	RGB from different sensors	CNN segmentation + CNN regression	Multi-objective regression (Fresh weight and height) by a hybrid CNN framework (UNet + ResNet50), fusing RGB features, segmented images and geometric elements.

### 4.2 Parameter analysis of FC layers

In this study, the image acquisition distance and scale factor were taken as the input geometric vector of the branch of the regression CNN, so that the regression network could adapt to the digital images acquired at different shooting distances. In order to explore the effects of the number of neurons in the fully connected layer of regression network, we conducted further comparative experiments. Concretely, the number of neurons in the fully connected layer after the fusion of two paths (denoted as C1) was tested, as well as the number of neurons in the fully connected layer adjacent to the branch input (denoted as C2).

Experimental results are used to analyze the value of parameters. From **Table 7**, it can be seen that the estimation accuracy reaches an optimal level with increasing number of neurons but fluctuates. However, when the number of neurons in the fully connected layer increases to 1024, the estimation accuracy drops due to the excessive number of parameters. Higher values for the number of neurons were not tested due to computing resources limitations. **Table 8** shows the experimental results for various settings of C2. Although different values of C2 have different effects on the R^2^ of height and fresh weight, the R^2^ of both traits outperforms other results by setting C2 = 64. In conclusion, the experimental results prove that 512 and 64 are better choices for parameters C1 and C2, respectively.

**Table 7:** Regression error statistics of the proposed method for different C1 settings.

Parameter setting	SH			SFW		
C1	MAE(g)↓	NRMSE(%)↓	R^2^ ↑	MAE(g)↓	NRMSE(%)↓	R^2^ ↑
128	0.644	5.36	0.928	0.024	21.04	0.621
256	0.733	5.49	0.925	0.024	21.21	0.615
512	0.338	2.64	0.980	0.018	17.23	0.717
1024	0.788	5.88	0.914	0.024	22.68	0.560

**Table 8:** Regression error statistics of the proposed method for different C2 settings.

Parameter setting	SH			SFW		
C2	MAE(g)↓	NRMSE(%)↓	R^2^ ↑	MAE(g)↓	NRMSE(%)↓	R^2^ ↑
32	0.525	4.00	0.953	0.020	18.75	0.650
64	0.338	2.64	0.980	0.018	17.23	0.717
128	0.431	3.15	0.975	0.024	21.21	0.614
256	0.346	2.76	0.981	0.022	21.11	0.618

### 4.3 Limitations and future work

Although the results on the test set have proved that the proposed method is accurate and efficient in the seedlings image segmentation and growth traits estimation, the developed framework could still be improved potentially. First, the accuracy of plant height regression is higher than that of fresh weight regression because the images are captured from a single-side view of the rice seedling sample, which requires further mining the information provided by the lateral view to estimate the latter. Moreover, the input of the proposed framework is digital images of monocot plant seedlings, and its applicability to dicotyledonous plant seedlings needs to be further verified.

Future research will continue to collect more seedling images to expand the dataset, and not be limited to a single rice variety. To further improve the regression prediction accuracy of shoot fresh weight, images from multiple perspectives will be explored as input for the regression model. In addition, it is necessary to develop an automatic acquisition process of multi-view digital images of seedlings in the rice seedling factory, and the method in this study will be improved to adapt to the detection and non-destructive growth monitoring of single rice seedlings in complex backgrounds. Last but not least, the hybrid network is mainly composed of UNet and Resnet50, which can be easily deployed on edge computing devices or mobile phones. This means that the presented method is expected to be used for stationary automated phenotyping equipment in plant factories, as well as handheld mobile phenotyping equipment. When combined with mobile devices or integrated with an edge computing platform into vertical seedling factory facilities, this method has great application potential.

## 5 Conclusion

Rapid acquisition of morphological traits of rice seedlings can help to understand the growth status of rice seedlings, which is the key basis for intelligently controlling the environment of industrial seedlings and making lighting strategies. In this study, a semantic segmentation and growth-related traits estimation method for rice seedlings in a plant factory based on CNN and digital images was proposed, which could facilitate rice seedling growth monitoring. This method supports multiple intelligent terminals such as digital cameras or mobile phones as image acquisition devices and does not need to fix the image acquisition distance, so it is feasible in practical applications. The method was experimentally verified on the rice seedling dataset. The segmentation accuracy of rice seedling achieved an OA of 0.997, an F1 accuracy of 0.95 and an IoU accuracy of 0.91, and the estimated growth-related traits, such as plant height and shoot fresh weight, were also in good agreement with the measured values, with R^2^ values of plant height reaching 0.980 and NRMSE reaching 2.64%, R^2^ values of shoot fresh weight reaching 0.717 and NRMSE reaching 17.23%. The experimental results showed that the proposed method can accurately estimate the growth-related traits of rice seedlings using low-cost and easily accessible RGB digital images.

It can be concluded that the proposed method is a reliable estimation tool for growth-related traits at seedling stage of rice, which has good application potential in seedling growth monitoring. The accurate regression of growth-related traits can further provide support for scientific planting management and selection of varieties at seedling stage. In addition, because the proposed method is based on the common morphological characteristics of crops at seedling stage, it is promising to be used to realize the estimation of growth-related traits of other crops at seedling stage.

## Data availability statement

The raw data supporting the conclusions of this article will be made available by the authors, without undue reservation.

## Author contributions

ZY and DK conceived and designed the experiments. ZY and XT conducted the experiments, collected the data and wrote the manuscript. MD and XC collected the data and performed the statistical tests. PN provided the technical support. YL, YR, and DK read and revised the manuscript. All authors contributed to the article and approved the submitted version.
